# Assessment of the Biological Control Potential of Common Carabid Beetle Species for Autumn- and Winter-Active Pests (Gastropoda, Lepidoptera, Diptera: Tipulidae) in Annual Ryegrass in Western Oregon

**DOI:** 10.3390/insects11110722

**Published:** 2020-10-22

**Authors:** Inga Reich, Casi Jessie, Seung-Joon Ahn, Man-Yeon Choi, Christopher Williams, Mike Gormally, Rory Mc Donnell

**Affiliations:** 1 Department of Crop and Soil Science, Oregon State University, Corvallis, OR 97331, USA; casi.jessie@gmail.com (C.J.); rory.mcdonnell@oregonstate.edu (R.M.D.); 2 Applied Ecology Unit, National University of Galway, Galway H91 TK33, Ireland; mike.gormally@nuigalway.ie; 3 USDA-ARS Horticultural Crops Research Lab, Corvallis, OR 97331, USA; seungjoon.ahn@msstate.edu (S.-J.A.); mychoi@ars.usda.gov (M.-Y.C.); 4 Department of Biochemistry, Molecular Biology, Entomology and Plant Pathology, Mississippi State University, Mississippi State, MS 39762, USA; 5 School of Biological and Environmental Sciences, Liverpool John Moores University, Liverpool L3 3AF, UK; C.D.Williams@ljmu.ac.uk

**Keywords:** conservation biological control, slugs, qPCR, molecular gut content analysis, *Nebria brevicollis*, *Noctua pronuba*, *Deroceras reticulatum*

## Abstract

**Simple Summary:**

Many studies have shown that ground beetles feed on different agricultural pests, but little is known about their species communities from US cropping systems. We assessed the biological control potential of the most common carabid beetle species in Oregon annual ryegrass grown for seed by investigating spatial and temporal overlap of the most common species with those of the most damaging autumn- and winter-active pests (slugs, caterpillars and cranefly larvae) and determined the number of field-collected specimens that had fed on the respective pests using molecular gut content analysis. Only the non-native *Nebria brevicollis* was abundant during pest emergence and tested positive for all three pest groups. While the other common carabid beetle species*—Agonum muelleri*, *Calosoma cancellatum* and *Poecilus laetulus*—were also found to have consumed pests, they were active only during spring and summer, when crop damage by pests is less critical. We also show that disk tilling did not affect any of the four common carabid beetle species and that only *N. brevicollis* was significantly associated with a vegetated field margin. This study contributes to expanding our knowledge on conservation biological control in a system where chemical pesticides are still the mainstay of control against invertebrate pests.

**Abstract:**

While carabid beetles have been shown to feed on a variety of crop pests, little is known about their species assemblages in US annual ryegrass crops, where invertebrate pests, particularly slugs, lepidopteran larvae and craneflies, incur major financial costs. This study assesses the biological control potential of carabid beetles for autumn- and winter-active pests in annual ryegrass grown for seed by: (a) investigating the spatial and temporal overlap of carabids with key pests; and (b) molecular gut content analysis using qPCR. Introduced *Nebria brevicollis* was the only common carabid that was active during pest emergence in autumn, with 18.6% and 8.3% of *N. brevicollis* collected between September and October testing positive for lepidopteran and cranefly DNA, respectively, but only 1.7% testing positive for slug DNA. While pest DNA was also detected in the guts of the other common carabid species*—Agonum muelleri*, *Calosoma cancellatum* and *Poecilus laetulus*—these were active only during spring and summer, when crop damage by pests is less critical. None of the four carabid species was affected by disk tilling and only *N. brevicollis* was significantly associated with a vegetated field margin. However, as its impact on native ecosystems is unknown, we do not recommend managing for this species.

## 1. Introduction

Ground beetles (Coleoptera: Carabidae) are one of the most common arthropods in agroecosystems and their ability to aid in biological pest control has been demonstrated for several groups of pests including slugs [1,2,3,4,5,6] and caterpillars [7,8]. However, most carabids are generalist predators and their diet can comprise a variety of invertebrates including insects, slugs or earthworms, as well as seeds [9]. Feeding experiments conducted under limited or no-choice conditions in the laboratory can hence be problematic, as the carabids might feed on select pests that they would not or only sporadically eat in the field, where there is a greater availability of alternative prey. A more accurate way to assess actual pest predation is molecular gut content analysis of field-collected carabids using either standard polymerase chain reaction (PCR) or quantitative PCR (qPCR), which has been shown to be even more effective in detecting DNA traces of prey items in predator guts [10]. This approach has been used successfully in many studies investigating predation by carabids [11,12,13] and also has the added benefit of being able to determine the timing of feeding incidences. This is of particular interest as pest control by generalist predators is usually most effective when the pest in question is just becoming active and the predator to prey ratio is highest [14,15]. The natural enemies are thus able to delay, dampen or even prevent exponential pest population growth in the crop [16,17,18] while they are unlikely to significantly impact pest populations and prevent crop damage once the pests have become established [19]. Despite evidence of the opportunistic feeding behavior of certain species of carabids [20,21,22], it has been shown that some pests are consumed even if their numbers are low [23,24,25,26]. A prerequisite for pest control potential is the sufficient abundance of generalist predators in the fields at critical times, and to increase their effectiveness, habitat favorability should also be considered [17].

Carabid assemblages in agricultural fields are not related to crop type but rather to the timing of cultivation measures and changes in phenology and resulting microclimate specific to the crop [27]. In general, soil cultivation, such as tilling, disturbs the vertical and horizontal distribution of soil biota and, depending on the type of cultivation, buries all or some of the crop residue [28] which can serve as a habitat for pests and beneficials alike [29,30]. Different studies show different impacts of soil cultivation on carabid abundance [31,32,33,34,35], depending, for example, on depth or timing of cultivation as well as on carabid species. Carabid beetle assemblages are also affected by size and isolation of agricultural patches and the presence of permanent landscape elements such as natural woodlands or field margins [36,37,38,39,40,41,42]. These can serve as important refuge habitats and overwintering sites for a variety of beneficial arthropods [43,44] and may contribute to enhanced pest regulation in neighboring fields [45,46].

While much research has been carried out on the role of carabid beetles as predators in agroecosystems, this is mostly restricted to Europe and there is less knowledge of carabid assemblages and their pest control potential from cropping systems in the United States (US). Annual ryegrass *Lolium multiflorum* L. (AR) is a major cash crop in Western Oregon, where it is grown for seed. It is seeded in late summer or autumn around the first rain of the wet season and grows well in poorly drained soils, which are not suited for perennial grasses. The crop is most susceptible to damage in autumn and early winter when it is in the seedling stage (i.e., before tillering occurs), during which time a number of common pest species including slugs (Gastropoda), cutworms (Lepidoptera: Noctuidae) and cranefly larvae (Diptera: Tipulidae) start to become active [47]. Slugs thrive in the mild and wet climate of Western Oregon and account for ~$60 million damage to the state’s $500 million grass seed industry annually [48]. Here, slugs are most active between September and June, when humidity and soil moisture are high and they aestivate in the ground or beneath residue during the hot dry summer [49]. Mating and egg laying by the most common species *Deroceras reticulatum* (Müller) can occur in autumn and spring [50], leading to a large proportion of neonate and juvenile slugs in spring and early summer. Slugs are usually nocturnal but can be found feeding by day during fog or rain and damage to crops can be especially extensive around weedy and grassy field margins which serve as critical habitat [50]. Several cutworm species can pose a threat to grass seed crops, including glassy cutworm *Cymodes devastator* (Bruce), black cutworm *Agrotis ipsilon* (Hufnagel) and winter cutworm *Noctua pronuba* Linnaeus [47]. The latter species, in particular, is of concern as it is cold tolerant and can feed on crops throughout the winter when temperatures are above freezing [47]; its largely night-active larvae are present in the soil between September and March [51]. As this species was only discovered in Oregon in 2001, little is known about the impact of natural enemies. The larvae of the European cranefly *Tipula paludosa* Meigen and Common cranefly *Tipula oleracea* L. can be minor pests in non-irrigated autumn sown grasses such as AR and their activity increases usually after heavy rainfall, with stand loss occurring generally when plants are already weakened or stressed [47]. The present study takes a multi-step approach to assess, for the first time, the biological control potential of carabid beetles for these pests in AR in Western Oregon.

The objectives of this study were: (i) to identify the most commonly occurring carabid species in this system, and to investigate their spatial and temporal overlap with those of the key pests in AR; (ii) to quantify the predatory capacity of the selected carabid species on the key pests using qPCR-based prey identification of their gut contents; and (iii) to investigate the influence of field margin structure and disk tillage on the activity abundance of the common carabid species.

## 2. Materials and Methods

### 2.1. Study Sites and Sampling

This study was carried out in ten AR fields grown for seed in Linn County, Oregon, between 23 March 2018 and 20 June 2019. Five of these fields (‘experimental sites’, as tillage regime changed between the two years) were not tilled in August/September 2017 but tilled in August/September 2018, while the other five fields (‘control sites’, as tillage regime did not change between the two years) were tilled in both years (Table 1). Tillage at all sites was carried out with a disk ripper at 10–15 cm depth followed by secondary tillage with harrow and roller. AR was seeded in all ten sites in early autumn 2017 using a seed drill and fields were harvested the following June/July before being tilled in August/September and planted again in September 2018. Field sizes ranged from 15 to 81 hectares and soil types were moderately to poorly draining silt loams. More details on each site as well as usage of herbicide, fertilizer and other treatments can be found in Table S1.

Two parallel rows comprising five trapping points each were set up in every field. Every trapping point consisted of four pitfall traps surrounding one refuge trap (Figure S1), and one data logger was placed within each row. Rows 1 (‘edge traps’) and 2 (‘field traps’) were located at 10 and 70 m, respectively, from the closest field margin (Figure S1A). These distances were selected as Holland et al. [52] showed that species associated with the field margin were captured mostly within 60 m from the margin. A distance of 60 m between the traps was deemed sufficient to ensure sample independence, as Thomas et al. [53] demonstrated in a mark-recapture study that even after 30 days, *Pterostichus melanarius* Illiger rarely traveled further than 55 m from their release site. Within each tillage regime, the closest field margin of three sites consisted of a gravel strip adjacent to a road, which filled with water in the winter, while the closest field margin in the other two sites consisted of taller grasses, shrubs and trees, with the first few meters between the field and the margin usually sprayed with a herbicide. In sites C4, C5 and E4, the vegetation bordered a river, while it bordered a private garden in site E5 (Figure S2). Traps in site C1 had to be moved by approximately 500 m in September 2018 as the field was split and the initial trap location was planted with meadowfoam *Limnanthes alba* Benth but they stayed at the same distance to the closest field margin.

Pitfall traps (Figure S1C) were opened between 18:00 and 20:00 and collected the next morning between 06:00 and 08:00. Trap contents were emptied into labeled resealable plastic bags and placed immediately into a portable freezer containing dry ice to prevent further prey digestion as well as in-trap predation. Upon return to the laboratory, the bags were frozen until identification to species (carabid beetles, following [54]) or at least order (other invertebrates) level, after which samples were put into individual Eppendorf tubes containing 70% ethanol and stored at −20 °C. Refuge traps (Figure S1B) were deployed immediately after the pitfall traps were collected (to avoid providing a ‘slug hotspot’ for beetles to feed on right before pitfall trapping began) and were checked for slugs, slug eggs, caterpillars and cranefly larvae one week later between 09:00 and 18:00. After collection, all slugs and caterpillars were weighed and identified to species level. The grey garden slug, *Deroceras reticulatum*, was classified into four life stages based on weight (small 0–10 mg, intermediate 10–100 mg, large >100–200 mg and very large >200 mg) [55,56,57]. Cranefly larvae and slug eggs were counted in the field.

Sampling took place weekly and alternated between pitfall and refuge traps; the order in which fields were sampled was rotated to avoid sampling-time dependent bias. Not all fields could be sampled on every occasion due to farming operations in the fields, adverse weather conditions or flooding of certain sites. No pitfall sampling was undertaken in February 2019 due to very low overnight temperatures and persistent freezing rain. In total, there were between 23 and 26 refuge trap sampling occasions and between 21 and 25 pitfall trap sampling occasions per field (Table S2).

Three soil samples were collected monthly between 31 October 2018 and 5 May 2019 at 10, 40 and 70 m into the field using a bulb planter (diameter 7 cm, depth 10 cm). They were transferred into resealable bags and immediately put in a cooler chest before being stored at 5 °C in the laboratory. The following day, soil samples were thoroughly mixed within the bags and 25 ± 0.2 g of each sample were weighed out into aluminum foil pans (20 cm × 9.5 cm × 6.3 cm) and put into a drying oven at 105 °C for 24 h, after which they were weighed again to determine the soil moisture content. Plant species and percentage ground cover of vegetation (grouped by vegetation height: small herb layer <0.5 m, large herb layer 0.5–1 m, shrub layer >1–4 m, and tree layer >4 m) were recorded along the field edges adjacent to the traps in June 2019 (Table S3).

### 2.2. Statistical Analyses

All statistical analyses were carried out in R 3.6.1 [58].

The soil moisture content of the three samples collected at each time point were compared within each site for each sampling occasion using the Kruskal–Wallis test. Following a non-significant result, the same data were used to compare the soil moisture content between all sites using the Kruskal–Wallis test followed by Dunn’s test adjusted for multiple comparisons with ‘bh’ [59] (Figure S3, Table S4).

To investigate the effect of field margin structure on the distribution of the most common carabid species, generalized linear mixed-effects models (GLMMs) were created using the *lme4* package [60]. Count data of each trapping point per site and negative binomial distributions were used for all species, as the data were overdispersed. They included either the random effect ‘site’ (categorical with ten levels, see Table 1) only (null model), the random effect plus either of the fixed effects ‘field margin’ (categorical with two levels: gravel and vegetation, Table 1) or ‘soil moisture’ (numerical as ‘mean water loss’, Table S4) or all three effects (full model). The latter effect was included to detect possibly false associations with field margin type, as all four fields with vegetation margin were also those with the lowest soil moisture. The models were compared using parametric bootstrapping (N = 10,000) with the package *pbkrtest* [61]. Adjusted R^2^ values were calculated using *rsq* [62].

To investigate the influence of disk tilling on the activity density of the most common carabid species, a Before/After and Control/Impact (BACI) analysis was carried out using a two-way ANOVA with the factors ‘tillage regime’ (categorical with two levels: control and experimental) and ‘year’ (categorical with two levels: ‘year 1’ (23 March–13 June 2018) and ‘year 2’ (26 March–20 June 2019)) with the square root-transformed total number of specimens per site.

### 2.3. Molecular Gut Content Analysis

A total of 681 guts were dissected from the four most commonly found carabid species, considering both overall abundance and occurrence at eight or more sites (Table S5). Dissection of the crop and proventriculus was carried out in a sterile Petri dish using forceps that were sterilized with 70% ethanol and heat (using either an open flame or the ErgoSteri™ VT Glass Bead Sterilizer (PhytoTech Labs, Lenexa, KS, USA) 110 V at 250 °C) between each beetle. The guts were individually collected in 1.5 mL Eppendorf tubes and stored at −20 °C until DNA extraction.

The beetle guts were homogenized using a Kimble Kontes Pellet Pestle (DKW Life Sciences, Milville, NJ, USA) or bead beating and left until complete lysis in a water bath with frequent vortexing (usually approximately 5 h). Total DNA was extracted from 534 beetle gut samples (Table 2) in batches of 24 (23 samples and one blank extraction) using the DNeasy Blood and Tissue Kit (QIAGEN, Hilden, Germany), according to the manufacturer’s instructions. Guts that were damaged during dissection or that contained little or no food were not used for further analysis.

The gut extracts were screened using qPCR to identify feeding incidences on the commonly encountered pest groups in the fields: slugs, craneflies and caterpillars. Fewer gut samples were screened for craneflies compared to the other pests as larvae were only observed in the fields between December and May. Due to the variety of slug, caterpillar and cranefly species in the fields and our focus on pest groups rather than individual pest species, class- (Gastropoda), order- (Lepidoptera) or family (Tipulidae) specific primers were selected (GastNLSf1/Sr1 [63], Tip-gen-S267/A268 and Lep-gen-S274/A275 (both [64])) after tests confirmed the amplification of target and non-amplification of non-target species which were commonly found during this study (Table S6). While the GastNLS primer set would also amplify snail DNA, we did not see this as a problem as these gastropods can also cause significant economical damage to crops [65].

qPCR reactions (20 μL) contained 1X PowerUp SYBR Green Master Mix (Thermo Fisher Scientific, Waltham, MA, USA), 0.25 µM of each primer, 1 µL of template DNA and nuclease-free water. Reactions were run on an Applied Biosystems QuantStudio 3 or StepOnePlus Real-Time PCR System (Thermo Fisher Scientific, Waltham, MA, USA) using the following conditions for amplification: 95 °C for 10 min, followed by 40 cycles of 95 °C for 15 s and 60 °C for 1 min. To ensure that only the target product was amplified, a melting curve analysis was conducted after amplifications by heating samples to 95 °C for 15 s followed by 60 °C for 1 min and steadily increasing the temperature at 0.3 °C per min to 95 °C. It was confirmed that the PCR products dissociated between 87.5 and 88.1 °C (Gastropoda), 75.8 and 77.8 °C (Tipulidae) and 76.9 and 78.2 °C (Lepidoptera), respectively. Each 96-well plate contained 92 samples, two positive controls of target species DNA and two negative controls (non-target species and water).

## 3. Results

A total of 2442 carabids belonging to 36 species were collected from the pitfall traps during this study. The most abundant carabid species were *Nebria brevicollis* Fabricius (36.0%, non-native), *Calosoma cancellatum* Eschscholtz (13.3%), *Loricera foveata* LeConte (9.0%), *Poecilus laetulus* LeConte (8.8%), *Agonum muelleri* Herbst (6%, non-native) and *Amara longula* LeConte (5.8%), which together account for nearly 80% of caught specimens. While *N. brevicollis*, *C. cancellatum*, *L. foveata*, *P. laetulus* and *A. muelleri* were collected from at least eight of the ten sites, *A. longula* was found at six sites but was collected primarily (80.9%) from one site (Table S5). Due to this limited distribution, *A. longula* was not included in further analyses and *L. foveata* was excluded as it is a specialized collembola feeder.

A total of 1840 slugs belonging to five species were collected: the most abundant was non-native *D. reticulatum* (86.3%) followed by the Holarctic *Deroceras laeve* Mϋller (6.6%), of which both, native and introduced populations are found within the US, and non-native *Arion circumscriptus* Johnston (5.8%) (Table S7). A total of 1026 cranefly larvae (*Tipula* spp.) and 675 caterpillars (Lepidoptera) were also recorded during this study. The most abundant caterpillar was *N. pronuba* (89%) followed by *Cnephasia longana* Haworth (1.7%) and *Mythimna unipuncta* Haworth (1.6%).

### 3.1. Temporal and Spatial Overlap of Carabids and Annual Ryegrass Pests

**Figure 1:**
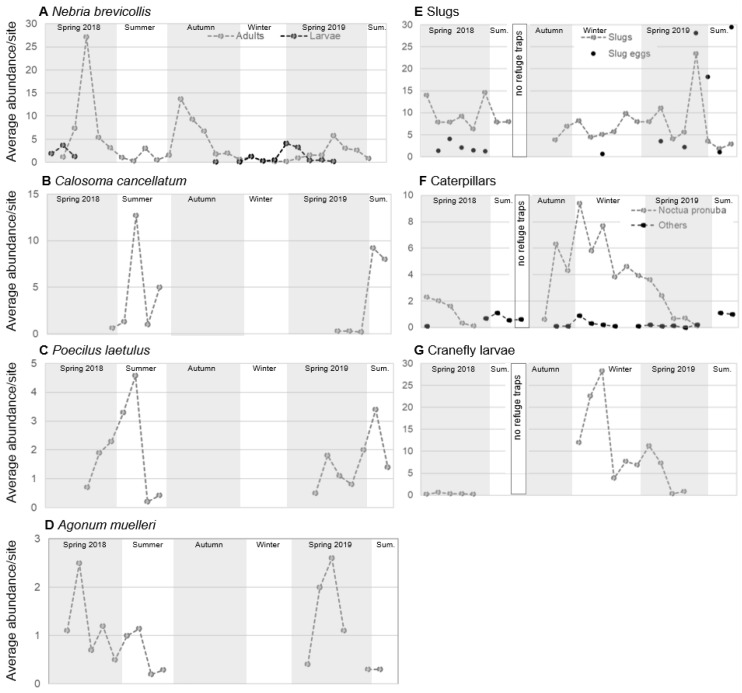
Average activity density per site for *Nebria brevicollis* (**A**), *Calosoma cancellatum* (**B**), *Poecilus laetulus* (**C**), *Agonum muelleri* (**D**), slugs (**F**), caterpillars (**F**) and cranefly larvae (**G**) throughout the sampling period (23 March 2018–20 June 2019). Refuge traps were not deployed between July and September 2018, caterpillars collected on this date were from pitfall catches. Spring: March–May, summer (Sum.): June–August, autumn: September–November, and winter: December–February.

The most abundant carabid species had highest activity densities in spring and summer, beginning from April (*A. muelleri* and *N. brevicollis*) or May (*P. laetulus* and *C. cancellatum*). Harvest operations started during the final week of June and halted sampling until the end of July or start of August (depending on the field), at which time only *C. cancellatum* was still captured in significant numbers (Figure 1A–D). *Nebria brevicollis* had a second activity phase in October and November with single specimens found throughout the winter as well. Additionally, its surface-active larvae were trapped between November and May, with a peak in larval activity in March and April (Figure 1A). Tenerals (newly emerged adults) were found between late March and early June, with a peak in late April/early May.

Slug activity was highest in May and relatively consistent for the rest of the year except during October 2018, when no slug activity was recorded (Figure 1E; due to summer aestivation no sampling for slugs took place between July and September). Eggs were found between April and May 2018 and between March and June 2019. While all life stages were found on nearly every sampling occasion, neonates were most abundant between April and May 2018 and again in June 2019, and juveniles were most abundant from May until December 2018 and a little less so from January to March 2019. Main activity for caterpillars was between November and March, when 83% of the total number were caught (Figure 1F). Cranefly larvae were observed only between December and May with 97% found between December and March (Figure 1G).

**Figure 2:**
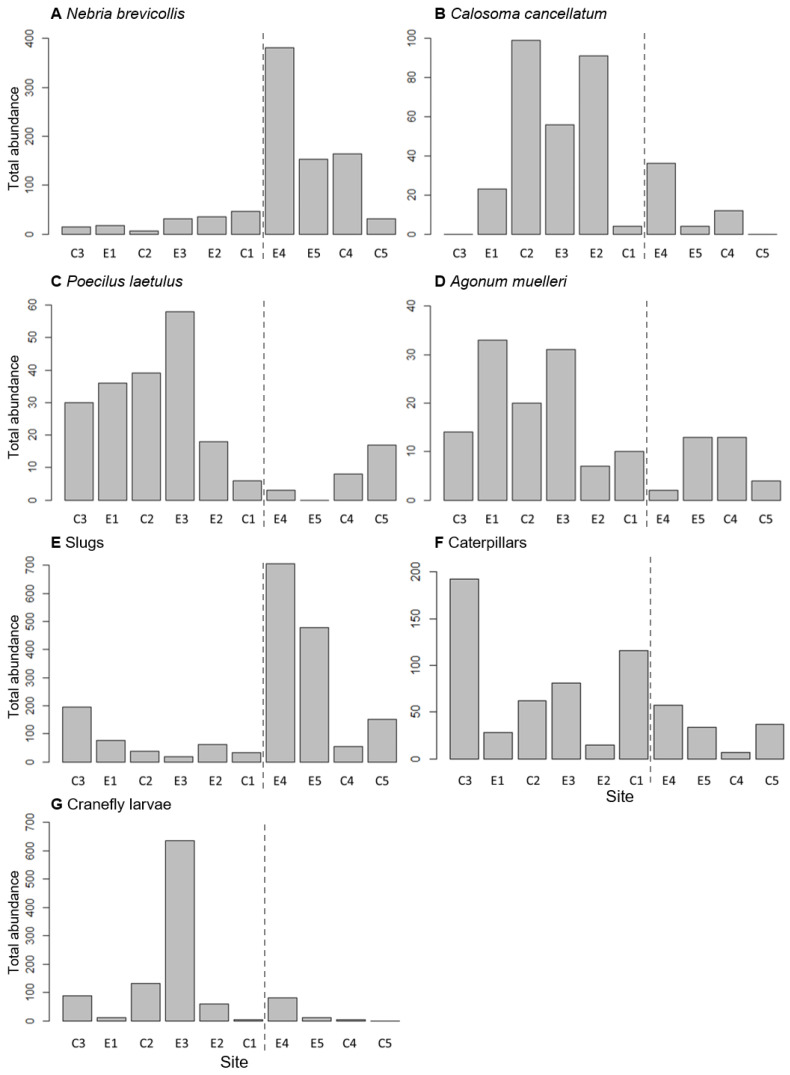
Total number of *Nebria brevicollis* (**A**), *Calosoma cancellatum* (**B**), *Poecilus laetulus* (**C**), *Agonum muelleri* (**D**), slugs (**E**), caterpillars (**F**) and cranefly larvae (**G**) caught per site over the duration of this study; sites are ordered by average soil moisture, from wettest (C3) to driest (C5) (see Table S4). Sites to the left of the broken line are those with a gravel margin, sites to the right have a vegetation margin.

*Nebria brevicollis* was found in all ten sites but was most commonly found in three of the four sites with a vegetation field margin and tended to be on the drier end of the soil moisture spectrum (Figure 2A). The other three common carabid species were found more commonly in the slightly wetter sites with gravelly field margins (Figure 2B–D). *Calosoma cancellatum* was completely absent from sites C3 and C5, while *P. laetulus* was absent from site E5. The largest number of slugs was found at sites E4 and E5 (Figure 2E), while caterpillars were most abundant at site C3 (Figure 2F) and cranefly larvae in site E3 (Figure 2G).

### 3.2. Impact of Field Margin and Disk Tilling on Carabid Species Distributions

The model containing the fixed effect ‘field margin’ was a significantly better fit than the model containing only the random effect ‘site’ for explaining the distribution of *N. brevicollis*, *P. laetulus* and *A. muelleri* and a better fit than those with ‘soil moisture’ or ‘field margin’ + ‘soil moisture’. Neither of the models containing any of the fixed effects was a significantly better fit than the model with only the random effect for *C. cancellatum* (Table 3). Association with the fixed effect was low in all cases and including ‘site’ produced a worse model fit than if it was omitted for *N. brevicollis* and *P. laetulus*.

**Figure 3:**
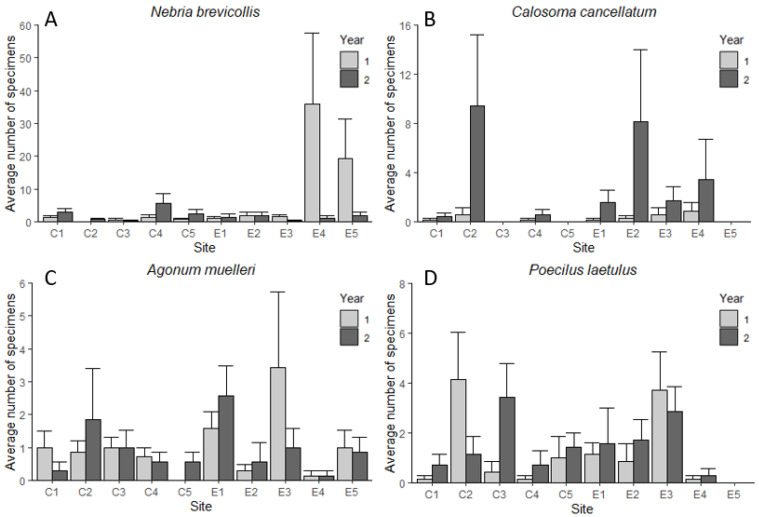
Average number + standard error of *Nebria brevicollis* (**A**), *Calosoma cancellatum* (**B**), *Poecilus laetulus* (**C**) and *Agonum muelleri* (**D**) in each site compared between year 1 (23 March–13 June 2018) and year 2 (26 March–20 June2019). The data are based on seven sampling occasions per year at each site.

When comparing the spring and summer data from 2018 and 2019, *N. brevicollis* numbers decreased sharply in two of the experimental sites (Figure 3), but no significant effects of ‘tillage regime’, ‘year’ or the interaction of both factors were found in the BACI analysis for this or any of the other three carabid species (Table 4).

### 3.3. Identification of Pest DNA in Carabid Gut Contents by qPCR

**Figure 4:**
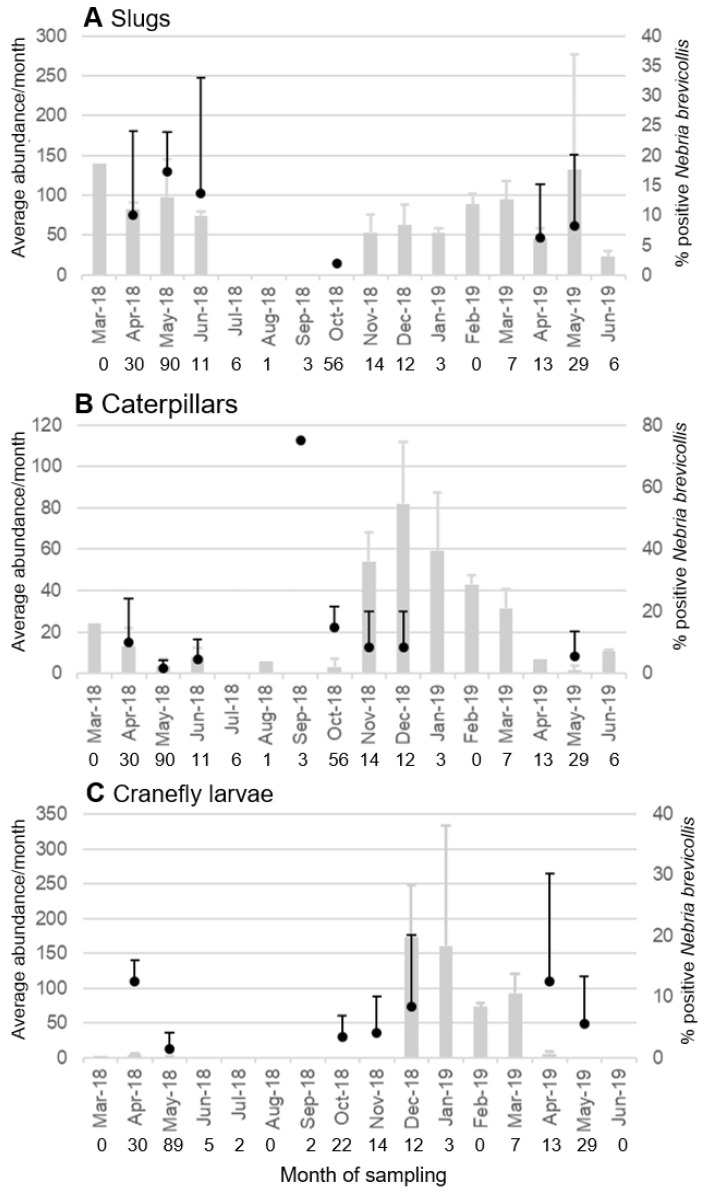
Seasonal change in the feeding incidences on the three pest groups by *Nebria brevicollis*. Average number + standard deviation (SD) of slugs (**A**), caterpillars (**B**) and cranefly larvae (**C**) collected per month (grey columns) and average percentage + SD of *Nebria brevicollis* that tested positive for the corresponding pest group (black dots). The numbers underneath the sampling months indicate the total number of *N. brevicollis* tested in each month.

Gastropod-specific DNA was amplified from three of the commonly found carabid species: *N. brevicollis* (26 out of 280 adult beetles (9.3%), and one out of 36 larvae (2.8%)), *P. laetulus* (four out of 99 beetles (4.04%)) and *A. muelleri* (one out of 51 beetles (1.96%)). All but one of these were from specimens collected between April and June; one *N. brevicollis* adult tested positive in October, when no slugs were found in the fields (Figure 4A). The majority of these beetles were collected from the two sites which had the largest number of slugs, 13 from site E4 and nine from site E5.

Screening for other pests in AR revealed that lepidopteran DNA was detected in 21 out of 280 (7.5%) *N. brevicollis*, one out of 44 (2.3%) *A. muelleri* and four out of 68 (5.9%) *C. cancellatum*. Fourteen out of 26 positive Lepidoptera samples were from *N. brevicollis* collected between September and December 2018, seven from specimens collected in April and May and five from carabids collected in June (Figure 4B). Only the three positive *N. brevicollis* that were trapped in November and December coincided with a period when caterpillars were found in large numbers. All *C. cancellatum* testing positive for Lepidoptera were collected in June, at a time when only a few caterpillars were detected using refuge and pitfall traps. Most positive testing carabids (nine) were collected in site C1, the site with the second largest total number of caterpillars (Figure 2F).

Tipulidae-specific DNA was detected from 14 out of 228 (6.1%) *N. brevicollis*, three out of 45 (6.7%) *A. muelleri* and three out of 61 (4.9%) *P. laetulus*. All but three of the 20 carabids that tested positive for Tipulidae were collected in April and early May of both years, at times when only a few or no cranefly larvae were observed in the fields, the other three were from *N. brevicollis* adults collected between October and December, two at times when no cranefly larvae were found (Figure 4C). The majority of all carabids testing positive for cranefly DNA (five) were collected from site E3, again the site where cranefly larvae were most abundant.

## 4. Discussion

The three pest groups that were monitored for this study were selected as they are active between autumn and spring and are known to cause serious damage to the emerging AR crop [47,49]. Thus, the identification of natural enemies that are active during the same time period is an important consideration for biological control. Of the common carabid species in AR, only *N. brevicollis* was found in large numbers during the early parts of this critical period, when it emerges from summer diapause to mate and lay eggs [66]. Abundance of this nocturnal carabid, which overwinters mainly in its larval stage [67], decreased in November but activity of *N. brevicollis* briefly overlapped with that of slugs and *N. pronuba* in late October/early November (Figure 1A,E,F). *Nebria brevicollis* larvae were found between December and April, with the highest activity between March and April followed by the emergence of the first tenerals around the same time, starting the spring activity phase of this species. Other common carabids in the fields also started to become active in late March/early April (*A. muelleri*, Figure 1D) or late April/early May (*P. laetulus*, Figure 2C), while *C. cancellatum* emerged in May (Figure 1B). Even if these three carabid species are not active during the time critical for crop damage in AR, slugs and their eggs and, to a lesser degree, caterpillars and cranefly larvae were still present in the fields and predation on these pests might still occur. This can lessen pest pressure the following autumn either indirectly by reducing the number of caterpillars and cranefly larvae metamorphosing into egg laying adults or directly by feeding on slug eggs and juvenile slugs that would otherwise have fed on emerging AR later in the year. The only distinct spatial overlap of carabid and pest abundances was between *N. brevicollis* and slugs, which were most abundant in the sites with vegetation margins.

Unlike with quadrats or soil sampling, the refuge traps we used in our study do not provide data on the actual density but rather the activity of the monitored organisms. This is dependent on conditions such as temperature, humidity or soil moisture (e.g., [68,69]), which can have different effects on activity depending on vegetation cover in the habitat [70,71]. While refuge traps were also reported to overestimate the abundance of surface-active species such as *D. reticulatum* [72] and underestimate juveniles [73] in comparison to soil sampling, Clements and Murray [74] found that ‘wadding square traps’, which are most similar to the refuge traps used in this study, caught more juveniles than saucer traps. The primary advantage of refuge traps over quadrats or soil samples is the comparably low sampling effort, which is crucial for large-scale experiments. Hence, they are frequently used to monitor slugs (e.g., [75,76,77]), but to our knowledge have not been used previously for cranefly larvae and caterpillars. As we were interested mainly in the activity periods of the pests and the differences in relative pest numbers between the sites, refuge traps were the ideal choice for this study.

*Nebria brevicollis* was the only common carabid in this study whose distribution was significantly associated with a vegetation margin. This species has traditionally been considered a woodland species associated with litter of deciduous trees and high soil moisture [67]. However, more recent publications report it to be rather eurytopic and abundant in a range of different habitats including agricultural grasslands and even urban areas [78,79]. While it occurred in all sites during this study, it was most abundant in those with a vegetation margin. It is likely that the vegetation field margins provide suitable habitat and refuge during harvest and post-harvest operations, which, in AR, also correspond with the timing of summer diapause and subsequent mating in *N. brevicollis*. However, its high mobility both as adult and larva [80] make it an effective colonizer of nearby fields that might offer suboptimal conditions for breeding or summer diapause. While the model containing the effect ‘field margin’ was also a significantly better fit than the null model for *P. laetulus* and *A. muelleri*, their activity densities in the different fields indicate an association with a gravel rather than a vegetation margin (Figure 2C,D). *Agonum muelleri* is described by some authors as a species that prefers open and dry habitats [81], while other authors classify this species as ‘hygrophilous’ [82] and list damp grasslands or even open woodlands as habitats [79]. No other studies have investigated the habitat preferences of *P. laetulus* to date and further work is needed to determine the ecological requirements of this species. ‘Field margin’ or ‘soil moisture’ did not explain the site preference of *C. cancellatum,* which is a species that is associated with open and dry country [54]. However, other soil characteristics such as grain size, pH and organic matter content (not measured in this study) could be important for this species as it was completely absent from site C3 (Figure 2B), the only site with slightly gravelly soil. In general, none of the associations with ‘field margin’ were strong (R^2^ < 0.21; Table 3) for any of the common carabid species, indicating that other factors not measured in this study are likely of greater importance.

Tilling can influence carabid beetles either directly by killing adults, larvae or pupae or indirectly, by altering biotic and abiotic habitat characteristics making the environment more or less suitable for different species [27,28]. None of the common carabid species in this study showed a significant response to disk tilling in August following the BACI analysis (Table 4), which is comparable with the findings of other studies. Holland and Reynolds [83] determined that tilling in October or February had no effect on the abundance of emerging *N. brevicollis* in fields of wheat stubble and undersown grass, and other studies even indicate that tilling might have a favorable impact on the species (summarized in [84]). Hatten et al. [85] state that, while *C. cancellatum* shows no consistent response to tillage, trap catches of this species were higher in conventional as opposed to no-till fields of peas, wheat and winter wheat. *Agonum muelleri* activity has been shown to be reduced by moldboard ploughing in July in a previous timothy/red clover field [86], but in Gareau et al. [87] it was significantly more abundant in full October tillage compared to reduced October tillage in cereal rye. While no studies have been carried out involving *P. laetulus*, related species *P. scitulus* was more abundant in conventional tillage systems compared to no till in spring wheat [88] and peas, wheat and winter wheat [85].

The large decreases in *N. brevicollis* numbers in sites E4 and E5 (Figure 3A) might be due to other effects: soil cultivation in this study took place at a time when *N. brevicollis* is in summer diapause, which is likely taking place in the field margin—Kromp [27] refers to an unpublished manuscript, that mentions *N. brevicollis* moving to the permanent boundary strip and woodland edge of the field during soil cultivation and seedbed preparation. If tilling had a direct mortality effect, it should have impacted the number of specimens found in autumn and subsequently also the number of larvae. However, activity density of the autumn 2018 population of *N. brevicollis* was high in site E4 and the number of larvae trapped the following spring was similar to those of the previous year. It is rather more likely that the larvae, all of which were caught in the edge traps in site E4, were pupating in or close to the field margins and died when the adjacent river burst its banks in early April 2019. Only one larva was caught at site E5 in spring 2018, followed by many adults. The autumn captures for *N. brevicollis* at this site were low, only two specimens were trapped in late October followed again by only a few larvae caught in winter 2018/spring 2019. This indicates that the species primarily inhabits the field margin at this site and only sporadically ventures into the field. This is supported by the finding that, unlike in site E4 where *N. brevicollis* was also found in large numbers in the field traps, nearly 90% of the captures at site E5 were from the edge traps. Why significantly fewer adults were captured at this site in the second year is unclear, but conditions might have been more suitable in the adjacent field margin and garden than within the field.

The majority of feeding incidences on gastropods as established by qPCR was accounted for by *N. brevicollis*, which is plausible given the temporal and spatial overlap between this beetle species and slugs. However, only one specimen tested positive for gastropod DNA during pest emergence in the autumn when slugs would pose the greatest problem. The remainder of the screened beetles that tested positive for Gastropoda were collected in spring/summer, possibly due to the higher abundance of smaller slugs at this time which are much easier to consume than larger adults [3] or due to a lack of more palatable prey. Feeding incidences on slugs were also highest in the two fields where these gastropods were most abundant and in the year in which they were present in especially high numbers, indicating opportunistic feeding events including scavenging rather than targeted predation. The overall feeding incidence was just under 10%, compared with 37% of *N. brevicollis* testing positive for slug DNA from a study in the United Kingdom [89], but according to the farmers of our study fields, pest pressure by slugs was much lower in the two years of the study compared to previous years, likely owing to the unusually warm and dry autumn weather in 2017 and 2018. Thus, slug numbers in the fields and associated feeding incidences by carabids might generally be higher than those recorded during the current study. Lower feeding rates for gastropods were observed for *P. laetulus* and *A. muelleri*, which is likely due to these beetles having lower abundances in the sites with high slug numbers (E4 and E5), and thus fewer encounters.

*Nebria brevicollis* also tested positive for Tipulidae and Lepidoptera DNA, and the beetles were observed feeding on live mature *N. pronuba* caterpillars as well as *Tipula* spp. larvae in the laboratory. Mair and Port [4] reported that *N. brevicollis* readily fed on Diptera larvae and Seric Jelaska et al. [90] showed that 20% of specimens tested positive when screened for Lepidoptera DNA with PCR, compared to 7.4% in this study. However, when looking solely at the presence of positive feeding events in September and October when early season predation would be at its most beneficial as the predator to pest ratio is largest, it was found that 18.6% of the 59 screened *N. brevicollis*, tested positive for Lepidoptera. This indicates that caterpillars might be an important food source during the second activity phase of this carabid species. While no species-specific primers were used, *N. pronuba* was the only abundant caterpillar in the AR fields during the autumn and winter season. This study provides the first feeding record of carabid beetles on this relatively new introduced pest species. Both caterpillars and cranefly larvae were consumed during pest emergence in the autumn, even when they were not found under refuge traps. This indicates that consumption of these two prey groups is probably not just the result of opportunistic feeding, as simple chance encounters with the prey would be less likely.

Lepidoptera were also consumed by *C. cancellatum*, which is described as ‘purely predaceous’ [91] and one pair of beetles in captivity was reported to have killed between five and twelve *Noctua clandestine* larvae a day [92]. In addition, Gidaspow [93] stated that *Calosoma* spp. could kill approximately 300 caterpillars per season with even their newly hatched larvae being able to overcome caterpillars of considerable size. It is hence surprising that only a few individuals (5.9%) tested positive for Lepidoptera DNA. However, only a few caterpillars were detected in our sites during peak *C. cancellatum* activity. The same applies for *P. laetulus*—while this latter species as well as *A. muelleri* showed some potential for contributing to pest control in AR, this study is the first to investigate their predation on these pest groups; still more studies are needed to elucidate their feeding preferences. A limitation of molecular gut content analysis is that it cannot distinguish between predation, scavenging [94,95] or secondary feeding [96,97]. Additionally, not every positive qPCR result necessarily indicates that the prey was killed. While the latter issue is difficult to resolve, as even an exact quantification of the amount of prey being consumed cannot prove the death of that prey, the ability and/or likelihood of a generalist predator actually feeding on live rather than dead prey can be determined through feeding experiments. We carried out two different laboratory trials (at 21 and 16 °C) using 20 adult *N. brevicollis* that were starved for seven days, which showed the species neither consuming eggs nor live juvenile *D. reticulatum* (~0.05 g; Appendix A) while dead slugs were readily eaten on other occasions, even when beetles were not starved (Inga Reich, personal observation). Other laboratory studies on the predation of slugs by *N. brevicollis* also showed very limited feeding on *D. reticulatum*, with most being restricted to very small, injured or dead individuals or eggs [3,4,98,99]. However, Ayre [2] found that up to 80% of one-day-old slugs presented to *N. brevicollis* at an ambient temperature of 8 °C were killed but mortality rate of slugs dropped at higher temperatures. *Nebria brevicollis* larvae were also found to feed on slugs, with ambient temperature being less important [2] but only one of the 36 larvae tested in the latter study were positive. It is hence questionable whether the positive results herein show actual predation events, even though they mainly occurred at a time where many neonate slugs were present. On the other hand, *N. brevicollis* was observed to feed on both, live mature cranefly larvae and final stage *N. pronuba* caterpillars in the laboratory, demonstrating that the positive qPCR results for these pests might constitute actual predation events rather than scavenging or secondary predation. Another limitation of molecular gut content analysis is that digestion will break down the prey DNA in the predator gut as time progresses. Using qPCR technique, slug DNA could still be detected in 42 out of 47 *N. brevicollis* even after 24 h after feeding on ~0.001 g tissue (Appendix B).

The next step in investigating the impact of commonly occurring carabids in AR fields should be to quantify the effect of predation by manipulating pest and predator densities in field cage experiments [100,101,102]. This is essential as Firlej et al. [13] found that despite a high incidence of feeding on soybean aphid *Aphis glycines* by *P. melanarius* (up to 33.7% of field-collected carabids screened positive for aphid DNA), a field cage experiment showed very little impact of carabid feeding on *A. glycines* population growth at moderate infestation levels.

## 5. Conclusions

Based on temporal overlap with all three common pest groups and on the results of the molecular gut content analysis in combination with feeding observations, *N. brevicollis* appears to be the most promising carabid for conservation biological control of cranefly larvae and caterpillars in the surveyed AR fields. Due to its autumn activity phase, this species could exert early season predation, which is critically important as it can delay pest population increase up to a point, after which the crop is less susceptible to damage. A potential problem is that *N. brevicollis* activity abundance decreases sharply in mid-November (Figure 1A) when *N. pronuba* and cranefly larvae are increasing in numbers (Figure 1F,G) and none of the larvae of *N. brevicollis*, which are active throughout the winter and early spring, were found to have consumed caterpillars or cranefly larvae.

While feeding incidences were slightly lower and the spatial and temporal overlap with any of the pest taxa was not as distinct, the other common spring-active species *P. laetulus* and *A. muelleri* and, to a lesser degree, the summer-active *C. cancellatum* still have some potential to reduce the populations of the pests at the times critical for crop damage. While none of the summer-active larvae of these three species were captured during this study, and neither *A. muelleri* or *P. laetulus* larvae have been reported to feed on any of the studied pest groups, those of *C. cancellatum* have been found to feed on caterpillars [92] and might contribute to conservation biological control of lepidopterans.

Disk tilling appeared to have no effect on the most common carabid species found in AR in this study. This was probably because these sites were under a no-till regime for only one year and it likely takes longer for carabid assemblages to reflect such reduced disturbance. Of the common carabid species, only the distribution of *N. brevicollis* was significantly associated with a vegetation field margin. The latter habitat is likely used by the species as a refuge during summer diapause and then as a source from where fields are re-colonized by either the larvae in the winter or the emerging tenerals the following spring. However, we do not recommend managing for this species, which is a relatively new introduction to Oregon because its impact on the native invertebrate fauna is not yet known [78]. The ecology and implications for the native carabid fauna of *N. brevicollis* in AR in Oregon will be addressed in a subsequent paper.

All raw data from this study that are not found in the Supplementary Materials have been uploaded to a public repository and can be accessed at https://doi.org/10.7267/p2677251h.

## Figures and Tables

**Table 1:** Tillage regime and field margin structure of the study sites. The site IDs will be used throughout the manuscript to identify each site.

	Experimental Sites					Control Sites				
Site ID	E1	E2	E3	E4	E5	C1	C2	C3	C4	C5
Tilled 2017	no	no	no	no	no	yes	yes	yes	yes	yes
Tilled 2018	yes	yes	yes	yes	yes	yes	yes	yes	yes	yes
Field margin	Gravel			Vegetation		Gravel			Vegetation	

**Table 2:** Number of guts per carabid species from which DNA was extracted and that were screened for pest DNA using qPCR.

Species	Number of Guts
*Agonum muelleri*	51
*Calosoma cancellatum*	68
*Nebria brevicollis* (adults)	280
*Nebria brevicollis* (larvae)	36
*Poecilus laetulus*	99
Total	534

**Table 3:** Results of the generalized linear mixed model analysis showing the best scoring model for each carabid species. Shown are the Χ^2^ and *p*-value of the comparison with the model containing only the random effect ‘site’ following parametric bootstrapping (N = 10,000) as well as the adjusted R^2^ values of the fixed effect (FE), random effect (RE) and full model (F).

Species/Pest Group	Best Model	Χ^2^	*p*	R^2^_adj FE_	R^2^_adj RE_	R^2^_adj F_
*Agonum muelleri*	AD = FM + S	4.662	0.031	0.08	0.13	0.21
*Calosoma cancellatum*	AD = 1 + S	n/a	n/a			
*Nebria brevicollis*	AD = FM + S	9.164	0.003	0.16	−0.03	0.13
*Poecilus laetulus*	AD = FM + S	7.702	0.006	0.11	−0.16	−0.05

AD = activity density, FM = field margin, S = site, *p* = *p*-value.

**Table 4:** Results of the BACI analysis with factors ‘year’ (Y) and ‘tillage regime’ (T) for each common carabid species.

Species		Df	*F* Value	Probability (>F)
*Agonum muelleri*	Y	1	0.019	0.891
	T	1	0.544	0.471
	Y:T	1	0.238	0.632
	Res.	16		
*Calosoma cancellatum*	Y	1	4.115	0.0595
	T	1	0.584	0.4558
	Y:T	1	0.466	0.5044
	Res.	16		
*Nebria brevicollis*	Y	1	1.008	0.3303
	T	1	2.431	0.1399
	Y:T	1	4.394	0.0523
	Res.	16		
*Poecilus laetulus*	Y	1	0.280	0.604
	T	1	0.026	0.873
	Y:T	1	0.004	0.949
	Res.	16		

Df = degrees of freedom and Res. = residuals.

**Figure A1:**
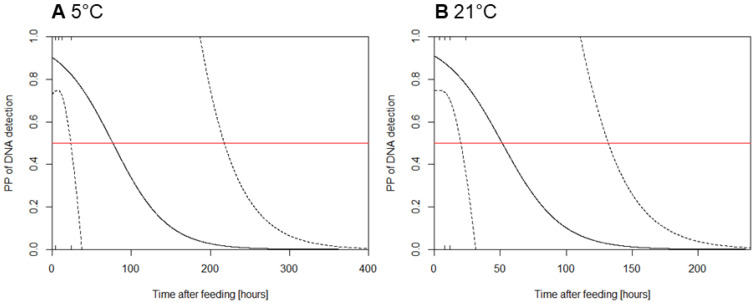
Predicted probability (PP, **solid line**) and 95% confidence intervals (**broken lines**) of slug DNA detection in the guts from *Nebria brevicollis* killed at different time intervals and kept at either 5 °C (**A**) or 21 °C (**B**) after feeding on *Deroceras reticulatum* cadavers. The red line indicates the point on the y axis where probability of DNA detection is 0.5. The small vertical lines at the top and bottom of the left side of each graph are the data from our study, indicating positive (located at y = 1.0) and negative (located at y = 0.0) qPCR results at each tested time slot (0, 4, 8, 12 and 24 h).

## Supplementary Materials

The following are available online at https://www.mdpi.com/2075-4450/11/11/722/s1, Figure S1: Details of the study setup and sampling equipment. Figure S2: Photograph of each field margin. Figure S3: Mean weight loss in grams of 25 g soil after 24 h at 105 °C for each site ± standard deviation on eight occasions between 31 October 2018 and 20 May 2019. Table S1: Field features and treatments used at each site between autumn 2017 and autumn summer 2019. Table S2: Sampling dates for pitfall and refuge traps at each site. Table S3: Plant species recorded in the field margins closest to the first row of traps in sites with a vegetation field margin (C4, C5, E4, E5) and general percentage of ground cover offered by each layer. Table S4: Mean weight loss in grams and standard deviation of all soil samples taken at each site and mean difference in weight loss in grams between sites. Table S5: Number of each carabid species collected by pitfall trapping between 23 March 2018 and 20 June 2019 at each site. Table S6: Species that were commonly found in the fields during this study and that were tested with the primer pairs GastNLSf1/GastNLSr1, Tip-gen-S267/Tip-gen-A268 and Lep-gen-S274/Lep-gen-A275. Table S7: Number of each slug species collected between 23 March 2018 and 19 June 2019 at each site.

## Appendix A. Feeding Trials

*Nebria brevicollis* were collected in the field two weeks prior to the experiment. They were kept in an incubator at 16 °C and a 12/12 light/dark cycle and maintained on frozen *Noctua pronuba* caterpillars. After being starved for seven days, they were put in a plastic container (height 12 cm, diameter 9 cm) lined with damp tissue paper and were presented with a juvenile *D. reticulatum* slug (~0.05 g) and/or two eggs from the laboratory colonies. The containers were left at 21 °C (trial 1) or 16 °C (trial 2) and at a 12/12 light/dark cycle for four days. The dead slug (Table A2) showed no signs of injury, and the chorion of the disturbed eggs was not damaged or ruptured (Table A1 and Table A2).

**Table A1:** Feeding trial 1 with 20 *Nebria brevicollis* kept at 21 °C and a 12/12 light/dark cycle and presented with two *Deroceras reticulatum* eggs each. The feeding trial was not checked on 2 June 2019.

Replicates	31 May 2019	1 June 2019	3 June 2019
1			
2			
3			
4			
5			
6			
7			
8			Eggs disturbed
9			
10			
11			
12			
13		Eggs disturbed	Eggs disturbed
14			
15			
16			
17		Carabid dead	trial ended
18			
19			
20			

**Table A2:** Feeding trial 2 with 20 *Nebria brevicollis* kept at 16 °C and a 12/12 light/dark cycle and presented with two *Deroceras reticulatum* eggs and a juvenile *D. reticulatum* (~0.05 g) each. The feeding trial was not checked on 12 June 2019.

Replicates	10 June 2019	11 June 2019	13 June 2019
1			
2			
3		1 slug hatched	
4			
5		1 slug hatched	
6			
7			Eggs disturbed
8			
9			
10			1 slug hatched
11			Eggs disturbed
12			Eggs disturbed
13			
14			Eggs disturbed
15			
16		Slug dead	
17			
18			
19			
20			1 slug hatched

## Appendix B. DNA Detection

*Nebria brevicollis* were collected in the field several weeks prior to the experiment. They were kept in an incubator at 16 °C and a 12/12 light/dark cycle and maintained on frozen *Noctua pronuba* caterpillars. A total of 50 adult *N. brevicollis* were provided with 0.01 g of slug tissue following a three day starvation period. Beetles were allowed to feed for 30 min and were killed by freezing at −20 °C in groups of five after 0, 4, 8, and 12 h. As three beetles did not consume slug tissue within the 30 min, only three (kept at 21 °C) and four (kept at 5 °C) were killed after 24 h. Even after 24 h post-feeding on slug tissue, DNA could still be detected in 6 out of 7 *N. brevicollis*. However, DNA detection was not determined for 5 out of 40 beetles in the other time intervals. Two generalized linear models (GLMs) with a binomial distribution were created in R, one for each temperature. The resulting regression equations were Logit *(*probability of DNA detection at 5° C) = 2.228 − 0.029 * time and Logit (probability of DNA detection at 21 °C) = 2.314 − 0.045 * time and median DNA detection times were calculated as 76.5 h at 5 °C and 51.5 h at 21 °C. Predicted detection probabilities for longer time periods post-feeding were calculated and plotted (Figure A1A,B), but the range of values within the 95% confidence interval is large.
