# Supplementary material for: Assessment of the Biological Control Potential of Common Carabid Beetle Species for Autumn- and Winter-Active Pests (Gastropoda, Lepidoptera, Diptera: Tipulidae) in Annual Ryegrass in Western Oregon
Source: Insects. 2020 Oct 22;11(11):722. doi: 10.3390/insects11110722 (PMC7690374; doi:10.3390/insects11110722)
Supplement: Supplementary file 1 [file insects-11-00722-s001.pdf]

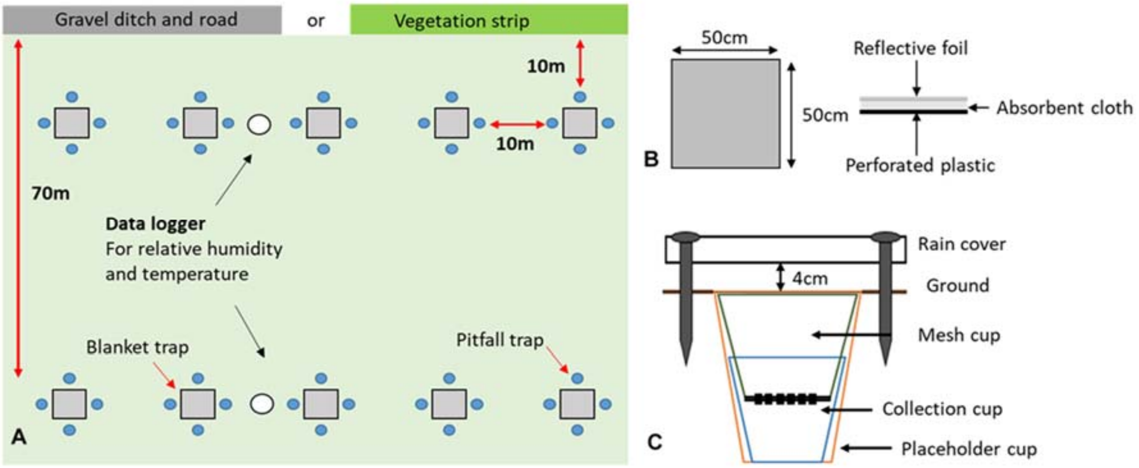

Figure S1. Details of the study setup and sampling equipment. (**A**) Schematic of sample site. The closest field margin consisted either of a gravel ditch that filled with water during the winter followed by a road (N = 3 per tillage regime) or of ground vegetation, shrubs and trees (N = 2 per tillage regime). Each trapping point consisted of four dry pitfall traps surrounding one refuge trap (0.25 m^2, De Sangosse). Two HOBO Pro v2 data loggers with radiation shield (ONSET) were installed in each field, one in each row. These were programmed to record temperature and relative humidity every 30 minutes. (**B**) Refuge trap (De Sangosse) from above and section of trap. **C** The dry pitfall traps were made up of three plastic cups: a placeholder cup (473 mL, Hefty) that was permanently buried into the soil and two nested collection cups. The top one (266 mL, 7.8 cm deep and 9.7 cm diameter wide at the top, Solo) had a mesh insert (0.42 cm mesh width) at the bottom, which prevented large carabids from reaching smaller prey items that could fall through the mesh into the bottom cup (266 mL, 9.3 cm deep, 7.9 cm diameter at the top, Solo). The mesh cup was painted with Fluon to prevent invertebrates from escaping and each trap was covered with a rain cover made of corriboard which was installed about 4cm above the trap and anchored in the soil with two masonry nails.

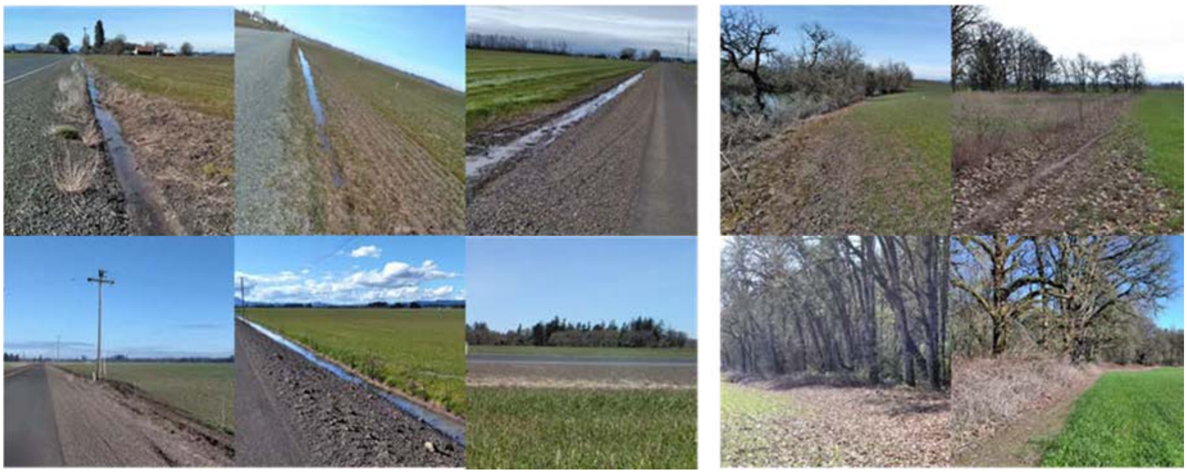

Figure S2. Photograph of each field margin. The pictures are grouped by treatment and structure of margin closest to the sampling plot. Top row: experimental fields E1, E2, E3, E4, E5; bottom row: control fields C1, C2, C3, C4, C5.

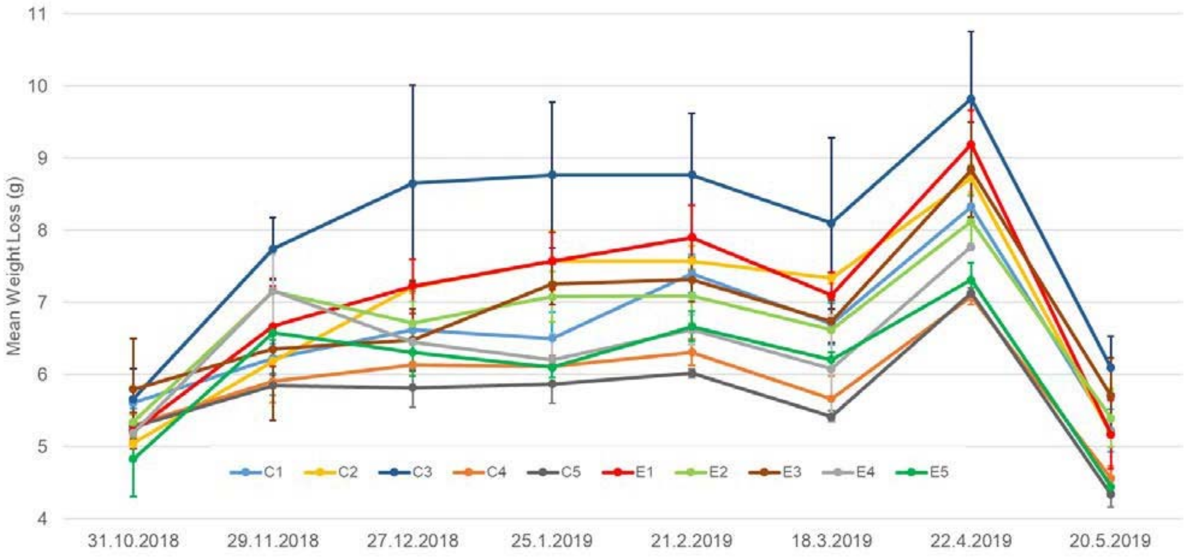

Figure S3. Mean weight loss in grams of 25 g soil after 24 h at 105 °C for each site ± Standard deviation on eight occasions between 31 October 2018 and 20 May 2019.

Table S1. Field features and treatments for each sites in the two years of the study.

	C1		C2		C3		C4		C5		E1		E2		E3		E4		E5	
**Years of Annual Ryegrass**	10		3		9		> 15		2		2		4		2		2		3	
**Previous crop**	Clover (?)		Tall fescue		Tall fescue		Meadowfoam		unknown		Meadowfoam		Meadowfoam		White clover		Wheat and oats		Wheat	
**Size (hectares)**	81		49		18		30		36		33		15		47		74		25	
**Max length/width (m)**	1410/674		789/607		645/416		561/536		625/775		875/417		389/380		882/530		1240/766		998/270	
**Soil types**	Woodburn 43%, Dayton 26%, Amity 22%, Willamette 5%, Concord 4%		Dayton 72%, Woodburn 22%, Amity 6%		Courtney 41%, Clackamas 31%, Whiteson 25%		Woodburn 67%, Amity 22%, Dayton 11%		Woodburn 66%, Dayton 18%, Concord 12%, Cloquato 4%		Woodburn 43% ,Dayton 35%, Amity 22%		Dayton 56%, Concord 37%, Amity 7%		Dayton 86%, Holcomb 10%, Amity 2%, Woodburn 2%		Dayton 60%, Amity 30%, Willamette 9%, Others 1%		Willamette 50%, Woodburn 24%, Dayton 20%, Amity 4%, Bashaw 2%	
**Season**	**2018/2019**	**2017/2018**	**2018/2019**	**2017/2018**	**2018/2019**	**2017/2018**	**2018/2019**	**2017/2018**	**2018/2019**	**2017/2018**	**2018/2019**	**2017/2018**	**2018/2019**	**2017/2018**	**2018/2019**	**2017/2018**	**2018/2019**	**2017/2018**	**2018/2019**	**2017/2018**
Tilled	yes	yes	yes	yes	yes	yes	yes	yes	yes	yes	yes	no	yes	no	yes	no	yes	no	yes	no
**Slug damage**																				
	low	low	none	none	none	none	none	low	none	none	none	none	none	none	none	none	medium	none	none	medium
**Caterpillar damage**																				
	low	low	none	none	none	none	none	none	none	none	none	none	none	none	none	none	none	none	none	none
**Cranefly larvae damage**																				
	low	low	none	none	none	none	none	none	none	none	none	none	none	none	none	none	none	none	none	none
**Molluscicide**																				
Application																	16.11.2018			24.10.2017
Product																	**Deadline MP**			**Deadline MP**
Rate																	12lbs			10lbs
Entire field/ edge only?																	S and SWedges			entire
**Insecticide**																				
Application			14.11.2018																	
Product			**Baythroid**																	
Rate			2.8ounces																	
Entire field/ edge only?			edge																	
**Herbicide**																				
At sowing	October	October	26.9.2018	18.10.2017	September	August	October	October	28.9.2018	14.10.2017	2.10.2018	9.10.2017	16.10.2018	22.9.2017	15.9.2018	23.9.2017	19.9.2018	30.9.2017	19.9.2018	30.9.2017
Product	**Nortron**	**Nortron**	**Glystar**	**Ethofumesate**	**Axiom**	**Axiom**	**Axiom**	**Axiom**	**Gramoxone**	**Gramoxone**	**Axiom**	**Glystar Plus**	**Gramoxone**	**Axiom**	**Axiom**	**Axiom**	**Axiom**	**Ethofumesate**	**Axiom**	**Ethofumesate**
Rate	3 pints	3 pints	1 quart	3 pints	8oz	8oz	3 pints	3 pints	1 quart	1 quart	7 ounces	3 pints	1 quart	5 ounces	7 ounces	5 ounces	7 ounces	3 pints	7 ounces	3 pints
Entire field/ edge only?	entire	entire	entire	entire	entire	entire	entire	entire	entire	entire	entire	entire	entire	entire	entire	entire	entire	entire	entire	entire

Second autumn application			1.10.2018				October	October	1.11.2018	17.10.2017	5.11.2018	13.10.2017	2.11.2018	30.9.2017	1.11.2018	27.9.2017	7.11.2018	5.10.2017	5.11.2018	2.10.2017
Product			**Axiom**				**Nortron**	**Nortron**	**Nortron**	**Ethofumesate**	**Nortron**	**Ethofumesate**	**Nortron**	**Ethofumesate**	**Nortron**	**Glystar Plus**	**Nortron**	**Glystar Plus**	**Nortron**	**Glystar Plus**
Rate			7 ounces				3 pints	3 pints	3.75 pints	3 pints	3.75 pints	3 pints	3.75 pints	3 pints	3.75 pints	3 pints	3.75 pints	3 pints	3.75 pints	3 pints
Entire field/ edge only?			entire				entire	entire	entire	entire	entire	entire	entire	entire	entire	entire	entire	entire	entire	entire
Product								**Callisto**												
Rate								3oz												
Entire field/ edge only?								entire												
Third autumn application			8.11.2018											2.10.2017		18.10.2017				
Product			**Nortron**											**Gramoxone**		**Ethofumesate**				
Rate			3.75 pints											2 quarts		3 pints				
Entire field/ edge only?			entire											entire		entire				
Spring application			18.4.2019				early April	early April			22.4.2019		18.4.2019				17.4.2019			
Product			**MCPA**				**Banvel**	**Banvel**			**MCPA**		**MCPA**				**MCPA**			
Rate			15 ounces				8 ounces	8 ounces			1pint		1 pint				1 pint			
Entire field/ edge only?			entire				entire	entire			entire		entire				east edge only			
Product			**Huskie**				**MCP4 Ester**	**MCP4 Ester**			**Aim**		**Aim**				**Aim**			
Rate			1 pint				1 pint	1 pint			1 ounce		1 ounce				1 ounce			
Entire field/ edge only?			entire				entire	entire			entire		entire				east edge only			
Product							**Palisade**	**Palisade**												
Rate							1 pint	1 pint												
Entire field/ edge only?							entire	entire												
**Fungicide**																				
Application							March 302019	late April												
Product							**QuiltXL**	**QuiltXL**												
Rate							10-12 ounces	10-12 ounces												
Entire field/ edge only?							entire	entire												
**Fertilizer**																				

Autumn application	April	April	1.10.2018	17.10.2017	4.8.2018	September	October	October	22.9.2018	9.10.2017	2.10.2018	11.10.2017		22.9.2017	15.9.2018	23.9.2017	19.9.2018	30.9.2017	19.9.2018	30.9.2017
Product	Urea+Kmag	Urea+Kmag	13-39-0	13-39-0	14-23-105	0-0-60	10-20-20	10-20-20	13-39-0	13-39-0	13-39-0	13-39-0		13-39-0	13-39-0	13-39-0	13-39-0	13-39-0	13-39-0	13-39-0
Rate	400lbs	400lbs	100lbs	100lbs	121lbs	158lbs	150-200lbs	150-200lbs	100lbs	100lbs	100lbs	100lbs		425lbs	100lbs	100lbs	100lbs	100lbs	100lbs	100lbs
Spring Application				19.4.2018	April 2019	April 2018	early April	early April	29.3.2019	6.4.2018	1.4.2019	5.4.2018	29.3.2019	6.4.2018	29.3.2019	5.4.2018	3.4.2019	3.4.2018	10.4.2019	2.4.2018
Product				33-0-10	35-0-7-45	34-0-1-45	N+S	N+S	33-0-10	33-0-10	33-0-10	33-0-10	33-0-10-3-1.5	33-0-10	33-0-10	33-0-10	33-0-10	33-0-10	33-0-10	33-0-10
Rate				425lbs	406lbs	390lbs	145lbs	145lbs	425lbs	425lbs	425lbs	425lbs	425lbs	425lbs	425lbs	425lbs	425lbs	425lbs	425lbs	425lbs
**Grazed**																				
Time frame		Jan-Apr	Winter	Winter	Winter				Winter	Winter		Winter		Winter	Winter	Winter		Winter		
Breed		Sheep	Sheep	Sheep	Sheep				Sheep	Sheep		Sheep		Sheep	Sheep	Sheep		Sheep		
**Special events**																				
Anything else?								Tilled more than usual as fields were very dry												

Table S2. Sampling dates for pitfall and refuge traps at each site. Grey underlaid fields indicate that sampling took place. A = adverse weather conditions, F = field operations (harvest, spraying…), R = replacement sampling for previous week, W = waterlogged field, pitfalls flooded.

Pitfall Traps

	Date	C1	C2	C3	C4	C5	E1	E2	E3	E4	E5
	23/03/2018										
	04/04/2018										
	18/04/2018										
	02/05/2018										
	16/05/2018										
	30/05/2018										
	13/06/2018										
	26/06/2018	F	F			F					
	26/07/2018	F	F	F	F		F	F	F	F	F
	27/07/2018	F		F	F	F	F	F		F	F
	31/07/2018		F		F	F	F	F	F	F	F
	03/08/2018	F	F	F		F	F	F	F	F	F
	10/08/2018	F		F	F	F	F			F	F
	17/08/2018	F	F	F	F	F		F	F	F	
	22/08/2018	F	F	F	F	F	F	F	F		F
	27/09/2018		F		F	F	F	F	F	F	F
	10/10/2018	F		F			F				F
**Harvest and planting season**	17/10/2018										
	31/10/2018										
	14/11/2018										
	29/11/2018		A	A	A	A	A	A	A	A	A
	05/12/2018										
	21/12/2018		A	A		A		A			A
	15/01/2019						W				
	30/01/2019						W				
	13/03/2019						W				
	26/03/2019										
	17/04/2019										
	24/04/2019										
	08/05/2019										
	22/05/2019			A	A		A		A	A	A
	29/05/2019	R	R			R		R			
	05/06/2019										
	20/06/2019										

Refuge Traps

Date	C1	C2	C3	C4	C5	E1	E2	E3	E4	E5
23/03/2018										
04/04/2018										
09/04/2018										
24/04/2018										
10/05/2018										
24/05/2018	F									
11/06/2018										
21/06/2018	F									
09/10/2018	F		F			F				F
24/10/2018										
08/11/2020										
26/11/2018										
13/12/2018										
27/12/2018										
12/01/2019										
24/01/2019										
06/02/2019										
21/02/2019										
11/03/2019										

18/03/2019		
09/04/2019	F	
22/04/2019		
06/05/2019		
20/05/2019		F
04/06/2019		
19/06/2019		

Table S3. Plant species recorded in the field margins closest to the first row of traps in sites with a vegetation field margin (C4, C5, E4, E5) and general percentage of ground cover offered by each layer.

		C4				C5				E4				E5			
**Latin name**	**Common name**	**H1**	**H2**	**S**	**T**	**H1**	**H2**	**S**	**T**	**H1**	**H2**	**S**	**T**	**H1**	**H2**	**S**	**T**
Acer macrophyllum	Bigleaf maple				O												
Anagallis arvensis	Scarlett pimpernel	O	O														
Cirsium sp.	Thistle		R				O			R					O		
Claytona sibirica	Candy flower	R															
Conium maculatum	Poison hemlock		O	O							O						
Corylus cornuta	Beaked hazelnut				O												
Daucus carota	Wild carrot						O	R									
Echinocystis lobata	Wild cucumber	R	R			F	A	O		O	O	R					
Epilobium ciliatum	Fringed willowherb						R				R				R		
Fraxinus latifolia	Oregon ash				O												
Gnaphalium palustre	Western Marsh Cudweed	O															
Hedera sp.	Ivy	F															
Heracleum mantegazzianum	Hogweed										R						
Hypericum perforatum	St Johns Wort										R						
Madia sp.	Tarweed									R							
Mahonia aquifolium	Oregon grape					O	O	O									
Marahus oreganus	Oregon cucumber					O											
Matricaria sp.	Mayweed									O	O						
Poaecae	Grasses	O	O			O	O			F	O			A	A	O	
Polypodiopsida sp.	Bracken	O	O														
Polystichum munitum	Sword fern	O	O														
Prunus sp.	Fruit tree								F				R				F
Quercus sp.	Oak				O							R	R				A
Ranunculus sp.	Buttercup	O															

**Legend**		
**H1**	Herb layer 1	< 0.5m
**H2**	Herb layer 2	0.5-1m
**S**	Shrub layer	>1-4m
**T**	Trees	> 4m
**D**	Dominant	>75%
**A**	Abundant	51-75%
**F**	Frequent	26-50%
**O**	Occasional	11-25%
**R**	Rare	1-10%

Rosa nutkana	Nootka rose										R						
Rubus armeniacus	Himalayan blackberry	A	A	A		F	A	F		F	F	O					
Rubus ursinus	Trailing blackberry	O	O							R	R						
Rumex	Dock									R					O		
Salix	Willow								O								
Tellima grandiflora	Fringecup		R														
Thuja plicata	Western red cedar				R												
Verbascum blattaria	Moth mullein										R						
Vinca minor	Periwinkle		R														
General % ground cover by each layer	First 5 m	80%	0%	0%	0%	10%	80%	80%	40%	90%	60%	20%	10%	100%	100%	20%	0%
	After 5m	80%	40%	20%	100%	river	river	river	river	river	river	river	river	100%	0%	0%	90%

Table S4. Mean weight loss (MWL) in grams and standard deviation (SD) of all soil samples taken at each site (based on 24 samples: 8 sampling days, 3 samples per day apart from site E4: 7 sampling days, 3 samples per day = 21 samples; this site was adjusted for the missing sample day) and mean difference in weight loss in grams between sites. Bold numbers show significant differences between sites. * = $p < 0.05$, ** = $p < 0.01$, *** = $p < 0.005$ (Dunn's Test for multiple comparisons). Fields are sorted from wettest to driest from left to right and top to bottom.

Site	C3	E1	C2	E3	E2	C1	E4	E5	C4	C5
MWL	7.95	6.98	6.85	6.81	6.69	6.58	6.26	6.06	5.88	5.71
SD	1.55	1.35	1.24	1.08	0.94	0.97	1.01	0.94	0.73	0.77
**C3**	0	0.96	**1.09 ***	**1.13 ***	**1.25 ***	**1.36 ****	**1.68 *****	**1.88 *****	**2.06 *****	**2.23 *****
**E1**		0	0.16	0.2	0.32	0.43	0.75	**0.95 ***	**1.13 ****	**1.30 *****
**C2**			0	0.04	0.16	0.27	0.59	**0.79 ***	**0.97 ****	**1.14 *****
**E3**				0	0.12	0.23	0.55	**0.75 ***	**0.93 ****	**1.10 *****
**E2**					0	0.11	0.43	0.63	**0.81 ***	**0.98 *****
**C1**						0	0.32	0.52	**0.70 ***	**0.87 ***
**E4**							0	0.44	0.62	0.79
**E5**								0	0.18	0.35
**C4**									0	0.17
**C5**										0

Table S5. Number of each carabid beetle species collected by pitfall trapping between 23 March 2018 and 20 June 2019 at each site (34 sampling days, 21–25 occasions per site), the abundance of the dominant species at each site is highlighted in bold font.

Species	Total	E1	E2	E3	E4	E5	All E	C1	C2	C3	C4	C5	All C
Acupalpus meridianus	2					2	2						0
Agonum brevicolle	1			1			1						0
Agonum decorum	3		1				1	1		1			3
Agonum limbatum	5		1				1	1			2	1	4
Agonum melanarium	6						0				6		6
Agonum muelleri	147	33	7	31	2	13	86	10	20	14	13	4	61
Agonum piceolum	19				3		3			1	14	1	16
Agonum suturale	51	8	1	9			18		32			1	33
Amara aenea	10	10					10						0
Amara crassispina	1	1					1						0
Amara littoralis	6					6	6						0
Amara longula	141	**114**	9	5		1	129		1		11		12
Amara pallipes	2	2					2						0
Anisodactylus binotatus	18	10					10				5	3	8
Anisodactylus californicus	66	20		2	1		23	7	3	1	30	2	43
Anisodactylus sanctaecrucis	5	4		1			5						
Bradycellus californicus	2	1					1	1					1
Bradycellus congener	84	13	5	17	1		36	6	13	14	9	6	48
Calathus fuscipes	2		1				1					1	1
Calosoma cancellatum	325	23	**91**	**56**	31	4	205	4	**104**		12		120
Chlaenius sericeus	2					1	1				1		1
Clivina fossor	1						0			1			1
Dicheirus piceus	2						0	2					2
Harpalus nigritarsus	1	1					1						0
Loricera decempunctata	97	11	1	6	2	4	24	14	3	17	31	8	73
Loricera foveata	221	16	5	14	38	23	96	34	7	25	28	**31**	125
Microlestes nigrinus	38	1	1	3			5	2	26			5	33
Nebria brevicollis	880	17	36	30	**381**	**153**	617	**45**	7	15	**164**	**32**	263
Omus audoini	2					2	2						0
Platynus brunneomarginatus	57						0				57		57
Poecilus laetulus	215	40	18	**58**	3		119	6	39	**30**	4	17	96
Pterostichus algidus	3				1	1	2	1					1
Pterostichus melanarius	19		6	3	1	2	12				2	5	7
Scaphinotus marginatus	1						0				1		1
Stenolophus anceps	2				1		1	1					1
Stenolophus comma	2				1		1				1		1

Trechus obtusus	3				2		2	1					1
**Total no of specimens**	**2442**	**326**	**182**	**236**	**467**	**212**	**1423**	**136**	**255**	**119**	**391**	**118**	**1019**

Table S6. Species that were commonly found in the fields during this study and that were tested with the primer pairs GastNLSf1/ GastNLSr1 (P1, Jarman *et al.*, 2006), Tip-gen-S267/Tip-gen- A268 (P2), and Lep-gen-S274/Lep-gen-A275 (P3) (both from Sint *et al.*, 2014).

(Sub)class	Order	Family	Species	P1	P2	P3
Clitellata		Lumbricidae	*Lumbricus terrestris*			
Collembola		Entomobryidae	*Entomobrya sp.*			
Gastropoda		Agriolimacidae	*Deroceras laeve*	Yes		
			Deroceras reticulatum	Yes		
		Arionidae	*Arion circumscriptus*	Yes		
			Arion subfuscus	Yes		
Arachnida		Dictynidae	*Tricholathys sp.*			
		Gnaphosidae	*Zelotes sp.*			
		Linyphiidae	*Tennesseellum formica*			
			Tenuiphantes tenuis			
Insecta	Coleoptera	Carabidae	*Calosoma cancellatum*			
			Loricera decempunctata			
			Nebria brevicollis			
			Poecilus laetulus			
		Coccinellidae	*Coccinella septempunctata*			
			Hippodamia convergens			
		Staphylinidae	*Philonthus carbonarius*			
			Philonthus cognatus			
	Diptera	Fanniidae	*Fannia canicularis*			
		Muscidae	*Muscina levida*			
		Phoridae	*Diplonevra funebris*			
		Scathophagidae	*Scathophaga stercoraria*			
		Syrphidae	*Eupeodes sp.*			
		Tipulidae	*Tipula sp.*		Yes	
	Hemiptera	Aphididae	*Rhopalosiphum padi*			
	Lepidoptera	Noctuidae	*Mythimna unipuncta*			Yes
			Noctua pronuba			Yes
			Peridroma saucia			Yes
		Tortricidae	*Cnephasia longana*			Yes

Table S7. Number of each slug species collected between 23 March 2018 and 19 June 2019 at each site (24 sampling days, 22–24 occasions per site). The abundance of the most dominant species at each site is highlighted in bold.

Species	Total	E1	E2	E3	E4	E5	All E	C1	C2	C3	C4	C5	All C
Arion circumscriptus	106	0	0	0	35	57	92	13	0	1	0	0	14
Arion subfuscus	9	0	0	0	0	9	9	0	0	0	0	0	0
Deroceras laeve	121	**52**	25	3	9	0	89	0	**26**	4	0	2	32
Deroceras reticulatum	1588	25	**38**	**15**	**661**	**417**	1156	**25**	13	**190**	**55**	**149**	432
Prophysaon andersoni	16	0	0	0	6	0	6	0	5	0	1	4	10
**Total specimens**	1840	77	62	18	711	483	1352	38	44	195	56	155	488
